# Quantitative MRI comparison of early and late parenchymal injury after transcallosal vs. endoscopic approaches for third ventricle colloid cysts

**DOI:** 10.3389/fsurg.2025.1698632

**Published:** 2025-11-27

**Authors:** Ufuk Erginoglu, Laura Eisenmenger, Walid Elshamy, Cagdas Ataoglu, Stephanie A. Armstrong, Halli T. Olsen, Umid Sulaimanov, Jordan Ross, Alexey Samsonov, Huseyin Erdem Ak, Abdurrahman Aycan, Abdullah Keles, Burak Ozaydin, Mustafa K. Baskaya

**Affiliations:** 1 Department of Neurological Surgery, University of Wisconsin School of Medicine & Public Health, Madison, WI, United States; 2 Department of Radiology, University of Wisconsin School of Medicine & Public Health, Madison, WI, United States; 3 Department of Neurological Surgery, Faculty of Medicine, Ain Shams University, Cairo, Egypt

**Keywords:** brain edema, colloid cyst, endoscopic approach, interhemispheric approach, parenchymal injury, third ventricle, transcallosal approach

## Abstract

**Background:**

The interhemispheric transcallosal (ITA) and endoscopic approaches (EA) are established treatments for third ventricle colloid cysts (TVCCs); however, their relative parenchymal impact and the progression of associated MRI changes from early to late postoperative stages remain undefined.

**Objective:**

To compare early volumetric MRI findings after ITA and EA for TVCC resection and determine whether early parenchymal injury persisted on late imaging.

**Methods:**

Twenty-three patients (ITA, 13; EA, 10) with early and late postoperative MRI were retrospectively reviewed. Early T2/FLAIR hyperintensity volumes were segmented along the surgical tract (burr-hole tract subtracted in EA). DWI/ADC imaging assessed diffusion restriction. Late MRI evaluated gliosis, encephalomalacia, and parenchymal loss. Statistical, correlation, and sensitivity analyses assessed associations while adjusting for cyst size and hydrocephalus.

**Results:**

Early MRI hyperintensity volume was smaller after ITA than EA (349 ± 218 mm^3^ vs. 2,952 ± 2,084 mm^3^; *p* < 0.001). Diffusion restriction occurred in 7.7% of ITA and 50% of EA (*p* = 0.052). Gliosis, encephalomalacia, and parenchymal loss on late MRI were absent after ITA but present in 50% of EA cases (*p* = 0.007 each), with larger early volumes in EA associated with gliosis (*p* = 0.032), encephalomalacia, and parenchymal loss (*p* = 0.016 each). These associations persisted after adjusting for cyst size and hydrocephalus. Gross total resection occurred in 92% of ITA and 50% of EA cases (*p* = 0.039).

**Conclusions:**

Compared with ITA, EA produced larger early parenchymal injury, half of which persisted as structural abnormalities on late imaging, indicating more persistent radiologic change.

## Introduction

Colloid cysts of the third ventricle (TVCC) are rare, benign epithelium-lined cystic lesions that originate from embryonic paraphysis remnants (1–3). They represent 0.5%–2% of intracranial masses and 15%–20% of intraventricular lesions, most often located in the anterior superior third ventricle (3–7). Although histologically non-neoplastic, they may cause obstructive hydrocephalus by occluding the foramen of Monro, leading to elevated intracranial pressure, neurological deterioration, or sudden death (8–12). Symptomatic cysts are typically managed surgically, whereas small, asymptomatic lesions, particularly those <10 mm, are often observed with serial imaging (4, 5, 11). The decision to intervene in asymptomatic cases remains a common dilemma for neurosurgeons (8, 10, 13, 14).

The deep location of TVCCs adjacent to the fornix and critical draining veins poses technical challenges for safe resection (5, 10). Various surgical techniques have been developed for the resection of TVCCs, including the interhemispheric transcallosal approach (ITA) and the endoscopic approach (EA) (4, 8, 15). The ITA, historically considered the gold standard, offers high rates of gross total resection (GTR) and low recurrence (5, 6, 16). In contrast, the minimally invasive EA has gained popularity for its smaller incisions, shorter hospital stays, and lower perioperative morbidity, though often with lower GTR rates and potentially higher recurrence (2, 4, 5, 10, 11, 16, 17).

The surgeon's experience, preference, and cyst characteristics, such as size and location, influence the choice between ITA and EA. Despite multiple retrospective studies and meta-analyses comparing recurrence, complications, and functional outcomes, the comparative parenchymal impact of these approaches—especially when quantified on postoperative magnetic resonance imaging (MRI)—remains poorly understood (4, 5, 8, 10, 11, 13, 14, 18–23).

Surgical manipulation of cortical, callosal, and periventricular structures can produce acute postoperative changes detectable on MRI (5, 8, 9, 14, 18, 23, 24). On early postoperative MRI, T2-weighted fluid-attenuated inversion recovery (T2/FLAIR) hyperintensity along the surgical tract most often reflects vasogenic edema but may include a cytotoxic component when diffusion restriction is present. The extent of these early changes and their potential progression to late sequelae such as gliosis, encephalomalacia, or parenchymal loss has not been systematically defined in TVCC surgery. To our knowledge, no previous study has performed volumetric quantification of early T2/FLAIR changes after ITA vs. EA and correlated these with late structural findings.

This study aims to address this gap by quantitatively comparing early postoperative parenchymal changes after ITA and EA, measured by volumetric T2/FLAIR hyperintensity, and assessing their relationship to late structural MRI findings, including gliosis, encephalomalacia, and parenchymal loss. By integrating quantitative MRI analysis with longitudinal follow-up, we seek to provide an objective comparison of the parenchymal impact of each approach and to clarify the radiologic evolution from acute change to delayed structural injury.

## Materials and methods

### Patient population

This institutional review board-approved study (IRB 2019-0432) retrospectively analyzed 23 patients who underwent TVCC resection at the University of Wisconsin Hospitals & Clinics between 2005 and 2019. The cohort consisted of 13 patients who underwent the ITA and 10 who underwent the EA. Due to the study's retrospective nature, the institutional review board did not require informed patient consent.

Inclusion criteria required both an early postoperative MRI within 7 days of surgery and a late MRI at ≥3 months, each containing T1-weighted imaging with and without contrast, T2/FLAIR, and diffusion-weighted imaging (DWI) with corresponding apparent diffusion coefficient (ADC) maps. Patients were excluded if imaging quality was poor, if they underwent secondary operations, or if clinical data were incomplete.

### Surgical decisions and patient inclusion

Surgical decisions were based on symptom severity and cyst size. In this study, all included patients underwent surgery. Symptomatic patients, particularly those presenting with severe headaches and signs of hydrocephalus, were prioritized for surgery. Although asymptomatic patients with cysts smaller than 10 mm are typically considered for conservative management, those who underwent surgery, such as due to risk factors including cyst growth or potential future complications, were included in the study.

Procedures were performed by two attending neurosurgeons with subspecialty expertise in endoscopic and microsurgical techniques, respectively. The choice of approach was based on surgeon preference, cyst size and location, ventricular dilation, and other patient-specific factors.

### Study parameters

Demographic and clinical data were recorded, including patient presentation, sex, cyst diameter, preoperative symptoms, postoperative complications, timing of imaging, reoperation rates, length of hospital stay, and follow-up duration. Hydrocephalus was defined radiologically by an Evans ratio greater than 0.30, accompanied by clinical signs of elevated intracranial pressure, including headache, nausea, and cognitive decline. Postoperative complications were documented, including new-onset neurological deficits, infections, seizures, intracranial hemorrhage, and memory disturbances.

The extent of TVCC resection was evaluated intraoperatively based on the surgeon's assessment of cyst wall removal and postoperatively through radiological imaging. GTR was defined as the complete removal of the cyst wall without residual tissue, confirmed by postoperative imaging.

### Imaging analysis

Early and late postoperative MRIs were reviewed to assess both acute and delayed parenchymal changes. All MRI scans were performed using clinical 1.5T or 3T systems with standardized sequences, including T1-weighted, T2/FLAIR, and diffusion-weighted imaging with ADC maps. Imaging protocols remained consistent throughout the study period to ensure reliable volumetric and qualitative comparisons. For early imaging, volumetric analysis of T2/FLAIR hyperintensity was performed to quantify vasogenic edema. Manual segmentation of hyperintense regions was conducted on each axial slice, with volumes calculated by summing slice areas and multiplying by slice thickness. For ITA cases, the region of interest (ROI) encompassed hyperintensity along the surgical tract; for EA cases, the measured burr hole tract volume was subtracted from the total hyperintensity volume to isolate parenchymal changes. Diffusion restriction on DWI with corresponding low ADC was recorded as present or absent to identify cytotoxic edema/acute infarction.

Late postoperative MRI was evaluated for gliosis, encephalomalacia, and parenchymal loss using T2/FLAIR and T1-weighted sequences. The extent of resection, residual cyst, and recurrence were assessed using T1-weighted imaging before and after contrast administration.

### Operative technique

#### Endoscopic approach

**Figure 1:**
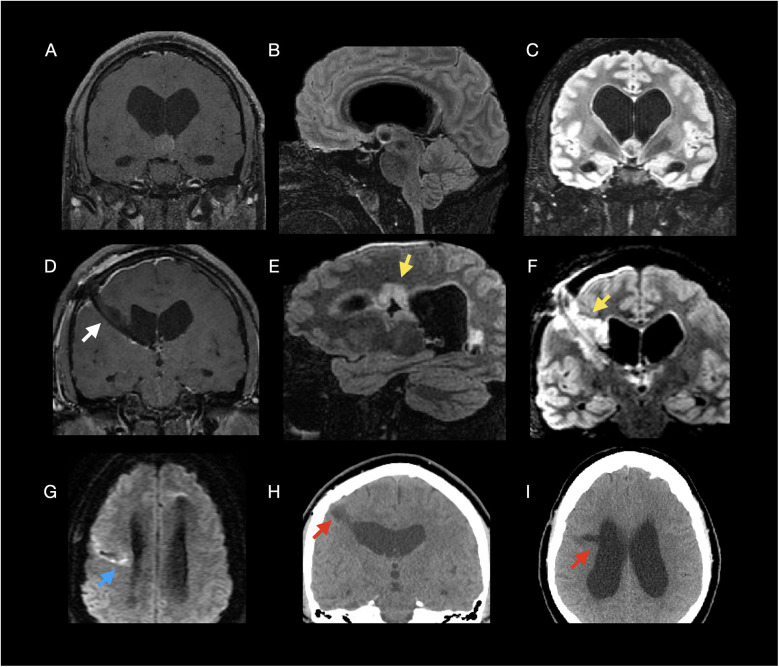
Multimodal imaging of a 24-year-old male with headache due to a symptomatic colloid cyst, treated with endoscopic resection. **Preoperative MRI (A–C):** Coronal **(A)** and sagittal **(B)** post-contrast T1-weighted, and coronal 3D T2-weighted FLAIR **(C)** images demonstrate a 1.6 cm homogeneously enhancing lesion in the anterior third ventricle at the foramen of Monro, consistent with a colloid cyst, causing obstructive hydrocephalus. **Postoperative day 1 MRI (D–G):** Coronal post-contrast T1-weighted **(D)**, sagittal **(E)** and coronal **(F)** 3D T2-weighted FLAIR, and axial DWI **(G)** show expected postoperative changes without residual enhancement, with hyperintensity surrounding the resection bed and a hypointense tract along the endoscopic trajectory consistent with postoperative edema and parenchymal injury; DWI **(G)** demonstrates hyperintensity consistent with diffusion restriction. **ThreE–Month folloW,Up CT (H,I):** Coronal **(H)** and axial **(I)** non-contrast CT images show chronic hypodense parenchymal loss extending toward the right lateral ventricle, consistent with postoperative sequelae. **Arrow key:** yellow arrows = postoperative hyperintensity **(E,F)**; white arrow = hypointense surgical tract **(D)**; blue arrow = diffusion restriction **(G)**; red arrows = parenchymal loss **(H,I)**.

**Figure 2:**
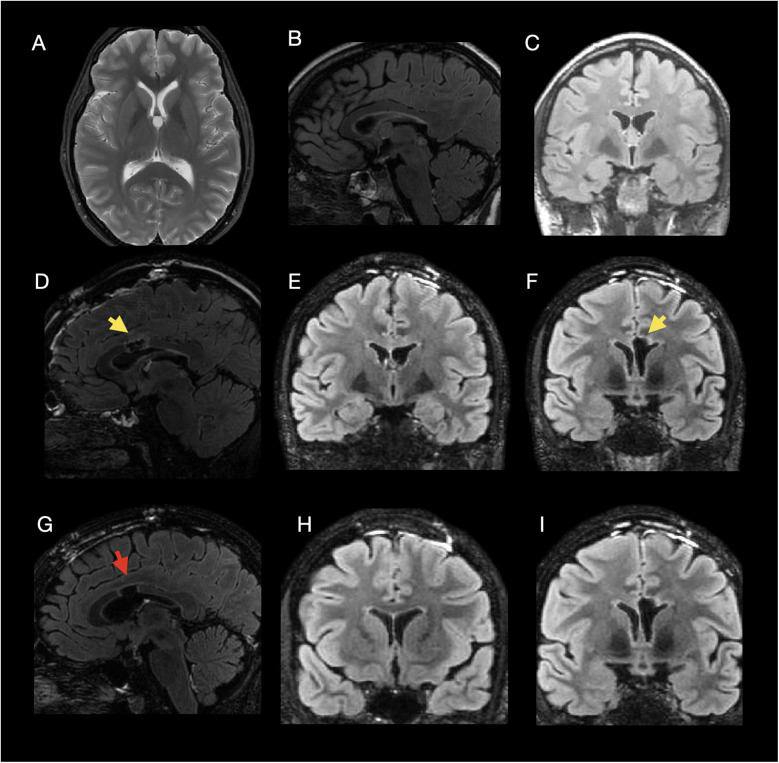
Multimodal imaging of a 27-year-old male with headache and ataxia due to a symptomatic colloid cyst, treated with endoscopic resection. **Preoperative MRI (A–D):** Axial **(A)** and sagittal **(B)** post-contrast T1-weighted, and sagittal **(C)** and coronal **(D)** 3D T2-weighted FLAIR images demonstrate a mildly hyperintense, well-circumscribed lesion measuring approximately 2.1 cm in maximum diameter, centered in the anterior third ventricle, consistent with a colloid cyst, causing obstructive hydrocephalus. **Postoperative day 1 MRI (E–H):** Sagittal **(E,F)** and coronal **(G,H)** 3D T2-weighted FLAIR images show expected postoperative changes without residual enhancement, along with moderate parenchymal hyperintensity surrounding the resection bed, consistent with postoperative edema and parenchymal injury. **FouR,Month folloW,Up MRI (I–K):** Sagittal **(I)** and coronal **(J,K)** 3D T2-weighted FLAIR images reveal no recurrence and demonstrate parenchymal volume loss and gliosis along the surgical tract. **TwentY,OnE–Month folloW,Up MRI (L):** Coronal 3D T2-weighted FLAIR image shows a new hyperintense lesion in the anterior third ventricle, consistent with interval cyst recurrence. **Arrow key:** Yellow arrows: postoperative hyperintensity **(E–H)**; red arrows: parenchymal loss and gliosis **(I–K)**; white circle: recurrent colloid cyst in the anterior third ventricle **(L)**.

For the endoscopic approach, patients were positioned supine with their heads secured in a Mayfield head clamp flexed at a 30-degree angle. Neuronavigation was routinely used to select the optimal entry point based on the individual's anatomy, cyst size, and trajectory to the foramen of Monro, which in some cases necessitated a relatively lateral approach. A 14-mm burr hole was drilled approximately 4 cm lateral to the midline and 8 cm posterior to the nasion, with neuronavigational guidance ensuring optimal access to the third ventricle. A 4 mm rigid endoscope with a 6.7 mm outer diameter peel-away sheath was used in all cases. After a small corticotomy was performed, a 30-degree endoscope was introduced into the frontal horn of the lateral ventricle via a ventricular catheter sheath under continuous warm saline irrigation. Key anatomical structures, including the septal vein, foramen of Monro, thalamostriate vein, anterior caudate vein, choroid plexus, and internal cerebral vein, were carefully identified and preserved during the procedure. The choroid plexus overlying the colloid cyst was coagulated, followed by cyst puncture and evacuation using suction and grasping forceps. Adherent cyst walls were excised with a morcellation device. External ventricular drains (EVDs) were placed selectively to manage elevated intracranial pressure, typically in patients with tight ventricles or hydrocephalus. While intraoperative hemorrhage is a general indication for EVD placement, it was not a factor in our series (Figures 1, 2).

#### Interhemispheric transcallosal approach

**Figure 3:**
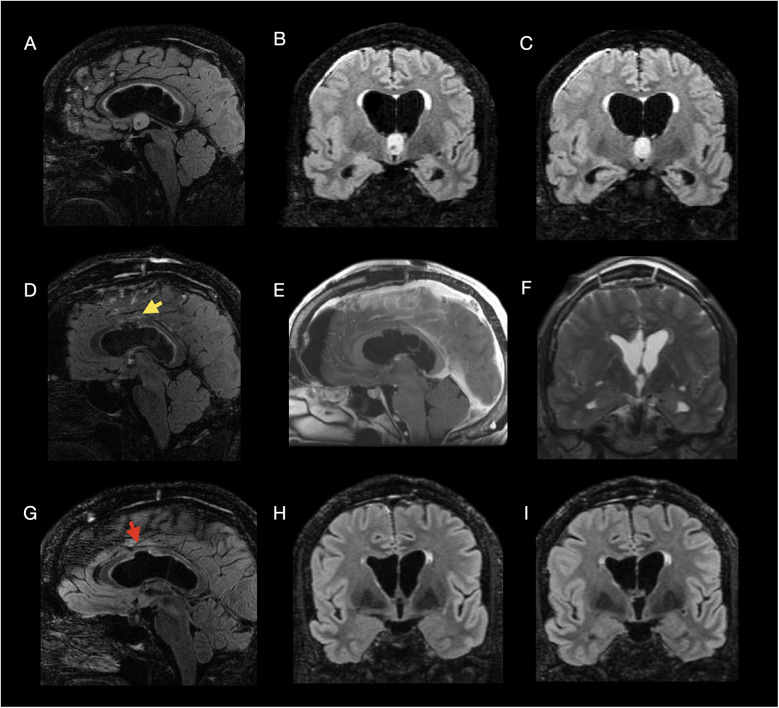
Multimodal imaging of a 64-year-old male with headache due to a third ventricular colloid cyst, treated via open interhemispheric transcallosal resection. **Preoperative images (A–C):** Sagittal **(A)** and coronal **(B,C)** 3D T2-weighted FLAIR images demonstrate a heterogeneously enhancing lesion measuring approximately 1.6 cm in maximum diameter at the superior margin of the foramen of Monro, consistent with a colloid cyst, causing moderate dilation of the lateral ventricles. **Postoperative day 1 MRI (D–F):** Sagittal 3D T2-weighted FLAIR **(D)**, sagittal post-contrast T1-weighted **(E)**, and coronal 3D T2-weighted FLAIR **(F)** images show expected postoperative changes without residual lesion, and minimal hyperintensity along the corpus callosotomy tract. **FouR,Month folloW,Up MRI (G–I):** Sagittal **(G)** and coronal **(H,I)** 3D T2-weighted FLAIR images show no recurrence and demonstrate the callosotomy defect without additional parenchymal tissue loss. **Arrow key:** Yellow arrow: postoperative hyperintensity **(D)**; red arrow: callosotomy defect **(G)**.

**Figure 4:**
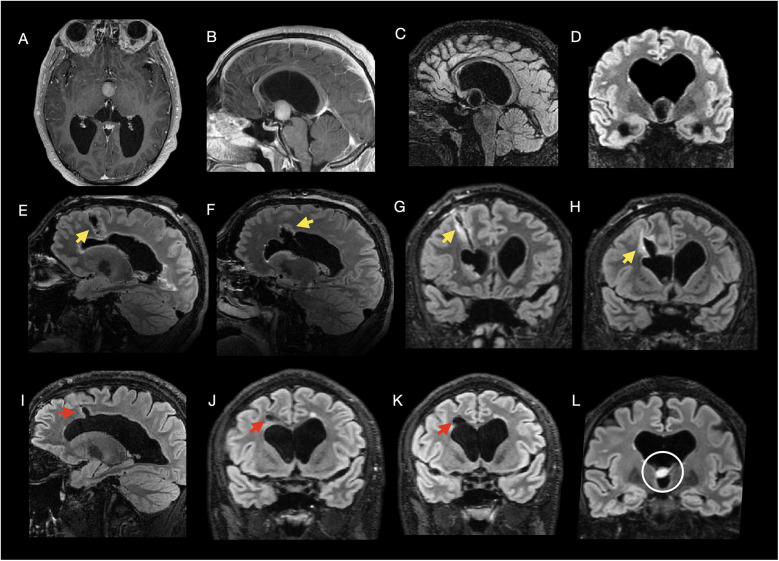
Multimodal imaging of a 22-year-old female with chronic headache due to a third ventricular colloid cyst, treated via open interhemispheric transcallosal resection. **Preoperative images (A–C):** Axial T2-weighted **(A)**, sagittal post-contrast T1-weighted **(B)**, and coronal 3D T2-weighted FLAIR **(C)** images demonstrate a smoothly contoured, non-enhancing hyperintense lesion measuring approximately 9 mm in diameter in the anterior third ventricle at the foramen of Monro, consistent with a colloid cyst, without ventricular enlargement. **Postoperative day 1 MRI (D–F):** Sagittal **(D)** and coronal **(E,F)** 3D T2-weighted FLAIR images show expected postoperative changes without definite residual enhancement, and focal hyperintensity around the corpus callosotomy tract and surgical corridor, without surrounding edema. **FouR,Month folloW,Up MRI (G–I):** Sagittal **(G)** and coronal **(H,I)** 3D T2-weighted FLAIR images show no residual lesion or recurrence and demonstrate the surgical callosotomy without surrounding hyperintensity or parenchymal tissue loss. **Arrow key:** Yellow arrows: postoperative hyperintensity **(D,F)**; red arrow: callosotomy defect **(G)**.

For the transcallosal approach, patients were placed supine with slight head elevation, with their heads secured in a Mayfield head clamp. A horseshoe incision was made behind the hairline, and paramedian burr holes were drilled lateral to the sagittal sinus to avoid vascular injury. The bone flap was raised, and the dura mater was opened to access the interhemispheric fissure. Dissection of the fissure proceeded cautiously, ensuring the protection of critical venous structures, such as bridging and internal cerebral veins. Upon identification of the corpus callosum, a callosotomy no larger than 1.5 cm was performed to access the lateral ventricle, allowing for controlled CSF drainage and brain relaxation. No fixed brain retractors were used; the interhemispheric corridor was maintained with gentle dynamic retraction using bipolar forceps and suction. Critical structures, including the septum pellucidum, fornix, choroid plexus, thalamostriate vein, and foramen of Monro, were carefully preserved throughout the procedure. A fenestration of the septum pellucidum was performed to connect the lateral ventricles, enhancing brain relaxation and access. The cyst wall was meticulously dissected from surrounding structures, including the fornix and internal cerebral veins. Hemostasis was achieved by coagulating the choroid plexus at the foramen of Monro, and the cyst was resected with care to ensure complete removal while avoiding damage to vital brain structures (Figures 3, 4).

### Statistical analysis

Continuous variables were compared between the ITA and EA groups using the independent samples *t*-test for normally distributed data and the Wilcoxon rank-sum test for non-normally distributed data. Categorical variables were compared using Fisher's exact test due to small sample sizes. Data are presented as mean ± standard deviation for continuous variables and as counts with percentages for categorical variables.

Associations between early postoperative T2/FLAIR hyperintensity volume and the presence of late MRI findings (gliosis, encephalomalacia, parenchymal loss) were evaluated within the EA group using independent samples *t*-tests. A two-sided *p*-value < 0.05 was considered statistically significant. Analyses were performed using IBM SPSS Statistics for Windows, Version 27.0 (IBM Corp., Armonk, NY) and R software, Version 4.0.3 (R Foundation for Statistical Computing, Vienna, Austria).

Additional correlation and subgroup analyses were performed to assess the relationship between cyst size, hydrocephalus status, and T2/FLAIR volume. Sensitivity testing was also conducted by excluding patients with cysts larger than 15 mm.

## Results

### Patient characteristics

The cohort included 23 patients (median age, 38 years; range, 14–79), with a higher mean age in the ITA group than the EA group (40.46 ± 17.25 vs. 29.8 ± 14.94 years; *p* = 0.128). Thirteen patients were male and 10 were female, with females comprising 31% of the ITA group and 60% of the EA group (*p* = 0.182). The most common presenting symptom was headache (ITA, 54%; EA, 70%). Hydrocephalus was present in 69% of ITA patients and 90% of EA patients (*p* = 0.227). Mean cyst diameter was significantly larger in the EA group (14.78 ± 3.93 mm) compared with the ITA group (11.08 ± 3.43 mm; *p* = 0.036). Placement of an external ventricular drain was more frequent in the EA group (70% vs. 23%; *p* = 0.027). No significant group differences were observed for other presenting features (Table 1).

**Table 1:** Presenting characteristics.

Characteristic	ITA (*n* = 13)	EA (*n* = 10)	*p* value
Age, years (mean ± SD)	40.46 ± 17.25	29.8 ± 14.94	0.128
Sex, female *n* (%)	4/13 (31%)	6/10 (60%)	0.182
Headache, *n* (%)	7/13 (54%)	7/10 (70%)	0.450
Loss of consciousness, *n* (%)	2/13 (15%)	2/10 (20%)	0.788
Nausea, vomiting, *n* (%)	0/13 (0%)	1/10 (10%)	0.343
Seizure, *n* (%)	1/13 (8%)	0/10 (0%)	0.337
Dizziness, *n* (%)	1/13 (8%)	0/10 (0%)	0.337
Memory loss, *n* (%)	0/13 (0%)	1/10 (10%)	0.343
Tremor, *n* (%)	0/13 (0%)	1/10 (10%)	0.343
Incidentally found, *n* (%)	2/13 (15%)	1/10 (10%)	0.713
Hydrocephalus, *n* (%)	9/13 (69%)	9/10 (90%)	0.227
Cyst diameter, mm (mean ± SD)	11.08 ± 3.43	14.78 ± 3.93	**0** **.** **036**
EVD, *n* (%)	3/13 (23%)	7/10 (70%)	**0** **.** **027**
ETV, *n* (%)	1/13 (8%)	2/10 (20%)	0.437

Bold values indicate statistical significance (*p* < 0.05).

EA, endoscopic approach; EVD, Extraventricular Drain; ETV, Endoscopic Third Ventriculostomy; ITA, interhemispheric transcallosal approach.

### Early and late MRI findings

Early postoperative MRI was obtained at a mean of 1.23 ± 0.44 days after surgery in the ITA group and 3.20 ± 2.39 days in the EA group (*p* = 0.013). The mean volume of early postoperative T2/FLAIR hyperintensity was significantly smaller in the ITA group (349.45 ± 218.48 mm^3^) compared with the EA group (2,951.51 ± 2,083.87 mm^3^; *p* < 0.001). On DWI/ADC, diffusion restriction was observed in 7.7% of ITA patients and 50% of EA patients, a difference that approached statistical significance (*p* = 0.052) (Table 2).

**Table 2:** Radiographic findings on early and late postoperative MRI.

Characteristic	ITA(*n* = 13)	EA (*n* = 10)	*p* value
Early MRI timing (days, mean ± SD)	1.23 ± 0.44	3.20 ± 2.39	**0** **.** **013**
Late MRI timing (months, mean ± SD)	3.31 ± 0.75	3.50 ± 0.97	0.492
T2 FLAIR Volume on Early MRI (mm^3^)	349.45 ± 218.48	2,951.51 ± 2,083.87	**<0** **.** **001**
Surgical tract volume, mm^3^	-	718.72 ± 638.25	–
Diffusion restriction on Early MRI, *n* (%)	1/13 (7.7%)	5/10 (50.0%)	0.052
Gliosis on Late MRI, *n* (%)	0/13 (0.0%)	5/10 (50.0%)	**0** **.** **007**
Encephalomalacia on Late MRI, *n* (%)	0/13 (0.0%)	5/10 (50.0%)	**0** **.** **007**
Parenchymal loss on Late MRI, *n* (%)	0 (0.0%)	5/10 (50.0%)	**0** **.** **007**

Bold values indicate statistical significance (*p* < 0.05).

EA, endoscopic approach; FLAIR, fluid-attenuated inversion recovery; ITA, interhemispheric transcallosal approach; MRI, magnetic resonance imaging.

On late postoperative MRI, gliosis, encephalomalacia, and parenchymal loss were absent in all ITA patients, whereas each was identified in 50% of EA patients (5 of 10), representing a statistically significant difference between groups (*p* = 0.007 for all comparisons) (Table 3).

**Table 3:** Association between early T2 FLAIR volume and late MRI findings in the EA group.

Late MRI Finding	Early T2 FLAIR volume—finding present (mean ± SD, mm^3^)	Early T2 FLAIR volume—finding absent (mean ± SD, mm^3^)	*p*-value
Gliosis	4,130.68 ± 2,202.28	1,772.34 ± 1,201.89	**0** **.** **032**
Encephalomalacia	4,236.18 ± 2,100.35	1,666.84 ± 1,110.55	**0** **.** **016**
Parenchymal Loss	4,236.18 ± 2,100.35	1,666.84 ± 1,110.55	**0** **.** **016**

Bold values indicate statistical significance (*p* < 0.05).

EA, endoscopic approach; FLAIR, fluid-attenuated inversion recovery; MRI, magnetic resonance imaging.

### Association between early and late MRI changes in the EA cohort

Within the EA cohort, larger early postoperative T2/FLAIR hyperintensity volumes were significantly associated with late structural changes. Patients with gliosis had a mean early hyperintensity volume of 4,130.68 ± 2,202.28 mm^3^ compared with 1,772.34 ± 1,201.89 mm^3^ in those without gliosis (*p* = 0.032). Similar associations were found for encephalomalacia (4,236.18 ± 2,100.35 mm^3^ vs. 1,666.84 ± 1,110.55 mm^3^; *p* = 0.016) and parenchymal loss (4,236.18 ± 2,100.35 mm^3^ vs. 1,666.84 ± 1,110.55 mm^3^; *p* = 0.016). These results indicate that early postoperative parenchymal changes are radiologically significant, as greater early change volumes are significantly associated with the development of late MRI abnormalities in EA patients (Table 4).

**Table 4:** Operative and clinical characteristics.

Characteristic	ITA(*n* = 13)	EA (*n* = 10)	*p* value
Intraoperative GTR, *n* (%)	12/13 (92%)	5/10 (50%)	**0** **.** **039**
Radiological GTR, *n* (%)	13/13 (100%)	8/10 (80%)	0.168
Weakness, *n* (%)	0/13 (0%)	0/10 (0%)	–
Transient memory loss, *n* (%)	2/13 (15.4%)	3/10 (30%)	0.440
Hemorrhage, *n* (%)	2/13 (15.4%)	3/10 (30%)	0.440
Infection, *n* (%)	0/13 (0%)	1/10 (10%)	0.343
Seizure, *n* (%)	2/13 (15.4%)	0/10 (0%)	0.165
Recurrence, *n* (%)	1/13 (7.7%)	2/10 (20%)	0.437
Reoperation, *n* (%)	0/13 (0%)	0/10 (0%)	–
Shunt dependence, *n* (%)	0/13 (0%)	1/10 (10%)	0.343
Hospital stays, days (mean ± SD)	4.92 ± 2.10	6.9 ± 3.03	0.098
Follow-up length, months (mean ± SD)	54.54 ± 29.44	33.60 ± 24.55	0.078

Bold values indicate statistical significance (*p* < 0.05).

EA, endoscopic approach; GTR, Gross Total Resection; ITA, interhemispheric transcallosal approach.

### Correlation and sensitivity analyses

Correlation analysis showed no significant association between cyst diameter and early postoperative T2/FLAIR hyperintensity volume (Pearson *r* = 0.15, *p* = 0.510). Similarly, there was no significant difference in T2/FLAIR volume between patients with and without hydrocephalus (*p* = 0.180, Wilcoxon test). After excluding patients with cysts larger than 15 mm, the EA group continued to demonstrate significantly higher T2/FLAIR volumes than the ITA group (*p* = 0.010).

### Operative and clinical outcomes

Intraoperative gross total resection (GTR) was achieved in 92% of ITA cases and 50% of EA cases (*p* = 0.039). Radiological GTR on early postoperative MRI was 100% for ITA and 80% for EA (*p* = 0.168). No new postoperative weakness was observed in either group. Transient memory loss occurred in 15.4% of ITA patients and 30% of EA patients (*p* = 0.440). Postoperative hemorrhage was documented in two ITA cases and three EA cases, and seizures occurred in two ITA patients but not in the EA group. Cyst recurrence occurred in one ITA patient (7.7%) and two EA patients (20%) (*p* = 0.437), and no reoperations were required. Mean hospital stay was shorter for ITA than EA (4.92 ± 2.10 vs. 6.90 ± 3.03 days; *p* = 0.098). Mean follow-up duration was longer for ITA (54.54 ± 29.44 months) than EA (33.60 ± 24.55 months; *p* = 0.078) (Table 4).

## Discussion

TVCCs are benign lesions but may cause life-threatening complications by obstructing cerebrospinal fluid (CSF) pathways, leading to hydrocephalus, elevated intracranial pressure, and, in rare cases, sudden neurological deterioration if untreated (5, 8–10, 12, 25). Symptomatic cysts generally require surgical intervention, and multiple approaches, including ITA and EA, have been developed over time. Each approach has its advantages and disadvantages, and debate remains regarding the optimal technique, particularly when considering not only clinical outcomes but also radiologically quantified parenchymal impact on postoperative MRI (1, 4–6, 11, 13). The objective of this study was to provide quantitative MRI-based measurement of acute and late parenchymal changes following ITA vs. EA, thereby contributing new imaging-based evidence to this ongoing discussion.

### Imaging analysis and Its implications

T2/FLAIR MRI sequences are well-suited for evaluating acute postoperative parenchymal changes, as they enhance lesion conspicuity near the ventricular margins by suppressing CSF signal (8, 26). In this study, early postoperative T2/FLAIR was used for volumetric quantification of vasogenic edema, and DWI with ADC mapping was used to identify the presence or absence of diffusion restriction, serving as a surrogate marker for cytotoxic edema or acute infarction. This combined radiologic approach provided a more nuanced characterization of early postoperative tissue changes for each surgical technique. Late postoperative MRI incorporated both T2/FLAIR and T1-weighted sequences to identify gliosis, encephalomalacia, and parenchymal loss, allowing for longitudinal correlation between early and delayed structural changes.

Although endoscopic approaches are widely considered minimally invasive due to smaller incisions and limited surface exposure, “minimally invasive” does not necessarily equate to reduced deep tissue disruption (6, 27). Our radiologic findings indicate that EA may be associated with more pronounced subcortical parenchymal changes. Rigid endoscopes traverse cortical and subcortical tissue directly, whereas the interhemispheric transcallosal approach follows a natural midline anatomical corridor without requiring cortical incision or the use of fixed retractors. These differences likely explain the significantly larger early T2/FLAIR hyperintensity volumes observed in the EA group (349.45 ± 218.48 mm^3^ vs. 2,951.51 ± 2,083.87 mm^3^; *p* < 0.001) and the higher incidence of diffusion restriction on early MRI (7.7% vs. 50%; *p* = 0.052), despite requiring a larger craniotomy (2, 4, 7, 11, 16, 20, 24). Moreover, late MRI abnormalities, including gliosis, encephalomalacia, and parenchymal loss, were absent in all ITA cases but present in 50% of EA cases (*p* = 0.007 for each). These findings are consistent with the expectation that transcortical entry may produce permanent tissue changes, although the extent and persistence of such radiologic abnormalities have not been widely quantified in the literature (5, 27–30)*.* Within the EA group, larger early T2/FLAIR hyperintensity volumes were significantly associated with the presence of each late abnormality (*p* = 0.016–0.032), underscoring the prognostic value of early postoperative imaging in predicting delayed structural injury. Collectively, these results provide objective, quantitative evidence that ITA may be less injurious to adjacent brain parenchyma than EA, both acutely and in the long term (Figures 1–4; Tables 2, 3).

### Endoscopic approach: is it truly less invasive?

The EA has been advocated for its small incision, shorter operative time, and potentially faster recovery (4, 11, 20, 25, 30–32). However, incorporating radiographic evidence of parenchymal injury offers a complementary perspective on the differential impact of each surgical approach. In our series, EA was associated with significantly larger early postoperative T2/FLAIR hyperintensity volumes and a higher rate of diffusion restriction than ITA, indicating more extensive parenchymal injury (Figures 1–4; Tables 2, 3). The presence of late sequelae exclusively in the EA cohort further supports the conclusion that reduced surface invasiveness does not necessarily equate to reduced deep brain injury.

Several factors likely contribute to this discrepancy. EA relies heavily on mechanical tissue manipulation and offers limited visualization, making precise dissection challenging, particularly within the confined spaces surrounding the third ventricle. Several technical factors may further explain these findings. EA often requires increased mechanical manipulation in a restricted operative corridor, with limited visualization and instrument maneuverability. Reliance on monopolar coagulation and suction during cyst wall dissection may increase the risk of collateral injury to periventricular white matter. These considerations are consistent with the observations of Isaacs et al. and Rangel-Castilla et al., who emphasized the technical constraints of endoscopic instrumentation (23, 29). Additionally, cyst wall adherence to the choroid plexus and internal cerebral veins can make complete and atraumatic removal particularly challenging via EA (3, 5, 8, 10, 18, 33–36).

In contrast, ITA, while requiring a larger craniotomy and more extensive cortical exposure, provides direct microscopic visualization, bimanual dissection, and greater surgical control (5, 6, 11, 31). These technical advantages may explain the significantly lower early T2/FLAIR volumes, lower incidence of diffusion restriction, and absence of late structural sequelae observed in the ITA cohort, suggesting that ITA may better protect deep brain parenchyma despite its more invasive surface approach.

### Gross total resection rates in the context of previous literature

Our findings are consistent with previous studies indicating that the ITA offers higher GTR rates than the EA (1, 3, 5, 6, 15, 35). Sheikh et al. demonstrated a 96% GTR rate for transcallosal approaches compared to 78.5% for EA in a meta-analysis of 1,278 patients (6). Similarly, Sethi et al. found that the transcallosal approach achieved a 96% GTR rate compared to 78.5% for EA (27). These studies underscore the advantages of the ITA in terms of achieving complete resection, which directly impacts recurrence rates. In our study, GTR was achieved in 92% of ITA patients, compared to only 50% in the EA group (*p* = 0.039) (Table 4). This significant difference highlights the limitations of the endoscopic approach, where the cyst wall often remains adherent to surrounding critical structures, such as the choroid plexus and internal cerebral veins, making complete resection more difficult (23, 37).

Beaumont et al. similarly reported that while the endoscopic approach is associated with a lower risk of immediate postoperative complications, it has a reduced GTR rate (36.4%) compared to microsurgical techniques (81%) due to the challenge of removing the cyst wall completely (10). These findings resonate with our data, emphasizing that the inherent limitations in visualization and instrument maneuverability in endoscopic procedures may explain why complete resection is less frequently achieved (11, 14).

### Complication rates and long-term outcomes

Despite greater parenchymal hyperintensity in the EA group, complication rates—including transient memory loss, postoperative weakness, hemorrhage, and infection—were comparable between groups (*p* > 0.05) (Table 4). This aligns with Alkhaibary et al. Horn et al. similarly found no significant difference in overall complication rates between microsurgical and endoscopic approaches for TVCC, despite Roth et al. suggesting potential morbidity and recovery advantages for EA (2, 11, 38, 39).

Hospital stay was shorter in the ITA group (4.92 ± 2.10 vs. 6.9 ± 3.03 days; *p* = 0.098), although not statistically significant. This contrasts with Beaumont et al., who reported reduced stays after EA (10). In our series, the longer hospital stay in the EA group was primarily related to EVD placement and the need for gradual weaning in patients with preoperative hydrocephalus. Moreover, a smoother recovery trajectory in the ITA group may have contributed to shorter hospitalization, potentially reflecting reduced parenchymal injury, as evidenced by lower T2/FLAIR hyperintensity volumes.

Another notable finding is the trend towards a longer follow-up duration in the ITA group (54.54 ± 29.44 months vs. 33.60 ± 24.55 months), though this difference was not statistically significant (*p* = 0.078) (Table 4). The longer follow-up in ITA patients could reflect a more rigorous postoperative monitoring protocol, potentially due to the expectation of higher complication rates with more invasive procedures. However, our data do not support this assumption, as the complication rates were similar across both approaches.

In addition to acute outcomes, our analysis incorporated late postoperative MRI to assess persistent parenchymal changes. Consistent with prior work highlighting the importance of extended follow-up for recurrence and delayed effects, we found that the greater early T2/FLAIR hyperintensity volumes in the EA group were frequently associated with chronic alterations on late imaging (6, 18, 23, 30). These radiologic findings suggest that the more extensive early parenchymal involvement after EA may have lasting structural consequences, even when not accompanied by overt clinical deficits.

### Cyst size and the role of CSF diversion

Cyst diameter was significantly larger in the EA group than in the ITA group (14.78 ± 3.93 mm vs. 11.08 ± 3.43 mm; *p* = 0.036) (Table 4). Larger cysts, often associated with more severe hydrocephalus, may necessitate preoperative CSF diversion (5, 7, 16, 26). In our series, 70% of EA patients required EVD compared with 23% of ITA patients (*p* = 0.027), consistent with Sethi et al., who noted a greater likelihood of preoperative diversion in EA due to more pronounced ventricular dilation (35). These factors may influence surgical selection toward EA despite its limitations and could contribute to the lower GTR rates and greater parenchymal injury observed in this group. Nonetheless, our late MRI findings indicate that a substantial proportion of EA-related parenchymal changes persisted beyond the acute phase, suggesting that these differences cannot be fully explained by cyst size, hydrocephalus severity, or the need for EVD, and that approach-specific factors play a significant role.

To address the potential influence of baseline differences, additional statistical analyses were performed. No significant correlation was found between cyst diameter and early T2/FLAIR hyperintensity volume (Pearson *r* = 0.15, *p* = 0.510), and T2/FLAIR volumes did not differ significantly between patients with and without hydrocephalus (*p* = 0.180). After excluding patients with cysts larger than 15 mm, the EA group continued to demonstrate significantly greater T2/FLAIR hyperintensity volumes (*p* < 0.010). These findings suggest that the surgical approach, rather than cyst size or hydrocephalus, was more strongly associated with postoperative parenchymal changes.

Although many imaging abnormalities were not associated with immediate neurological deficits, they may represent structural disruption with possible long-term implications. Our findings suggest that radiologic outcomes, such as gliosis and parenchymal loss, should be considered when defining invasiveness and may assist in counseling patients about the trade-offs between different surgical routes. These findings reflect institutions' experience and may not generalize to centers with different surgical expertise or resources. Clinical outcomes are likely influenced by both the technical nuances of each approach and the experience of the operating surgeon.

### Study limitations and future directions

A major strength of this study is the inclusion of both early and late postoperative MRI in all patients, enabling direct evaluation of the progression of parenchymal changes. The persistence of early postoperative T2/FLAIR hyperintensity into late imaging represents a significant radiologic finding, and its clinical implications merit further study. Nonetheless, the modest sample size limits statistical power and generalizability. Although both groups had long-term clinical follow-up, detailed neurocognitive and functional testing were not systematically performed. Future studies with larger cohorts and comprehensive neuropsychological assessment are needed to determine whether these radiologic changes translate into measurable cognitive or functional effects.

## Conclusion

This study challenges the prevailing belief that the EA is inherently less invasive than the ITA for TVCC resection, based on radiologic evidence of greater postoperative parenchymal changes following EA. Although often regarded as minimally invasive, the EA was associated with markedly greater early postoperative parenchymal hyperintensity on T2/FLAIR and more frequent diffusion restriction, a substantial proportion of which persisted on late MRI. The persistence of these findings indicates that acute alterations are not always transient and may represent lasting parenchymal changes. Consistent with prior literature, our study also demonstrated lower gross total resection rates with EA compared to ITA. Optimal ITA execution involves meticulous microsurgical dissection under high magnification, the use of neuronavigation to guide a small callosotomy and surgical trajectory, preservation of bridging and draining veins as well as pericallosal and callosomarginal arteries, gentle tissue handling, and transforaminal exposure via subchoroidal or suprachoroidal variants to protect the fornix and thalamostriate–septal venous complex, with controlled detachment of the choroid plexus from the foramen of Monro and septum pellucidum fenestration as indicated. When performed with ultramicrosurgical precision, these techniques can achieve high rates of complete resection while minimizing parenchymal injury, supporting the continued role of ITA as a preferred approach over EA in appropriately selected cases. While ITA may offer radiologic advantages in terms of reduced parenchymal changes, its successful implementation depends on meticulous microsurgical technique, and it should be interpreted within the context of individual surgeon expertise and institutional practice patterns.

## Data Availability

The original contributions presented in the study are included in the article/Supplementary Material, further inquiries can be directed to the corresponding author/s.
